# Genomic analysis of bluetongue virus episystems in Australia and Indonesia

**DOI:** 10.1186/s13567-017-0488-4

**Published:** 2017-11-23

**Authors:** Cadhla Firth, Kim R. Blasdell, Rachel Amos-Ritchie, Indrawati Sendow, Kalpana Agnihotri, David B. Boyle, Peter Daniels, Peter D. Kirkland, Peter J. Walker

**Affiliations:** 1 CSIRO Health & Biosecurity, 5 Portarlington Road, Geelong, VIC 3220 Australia; 2 School of BioSciences, The University of Melbourne, Parkville, VIC 3010 Australia; 3 CSIRO Australian Animal Health Laboratory, 5 Portarlington Road, Geelong, VIC 3220 Australia; 4 Virology Department, Indonesian Research Center for Veterinary Science, Bogor, West Java 16114 Indonesia; 5 Biosecurity Sciences Laboratory, 39 Kessels Road, Coopers Plains, Brisbane, QLD 4109 Australia; 6 Virology Laboratory, Elizabeth Macarthur Agriculture Institute, Woodbridge Rd, Menangle, NSW 2568 Australia; 7 School of Biological Sciences, University of Queensland, St Lucia, QLD 4067 Australia

## Abstract

The distribution of bluetongue viruses (BTV) in Australia is represented by two distinct and interconnected epidemiological systems (episystems)—one distributed primarily in the north and one in the east. The northern episystem is characterised by substantially greater antigenic diversity than the eastern episystem; yet the forces that act to limit the diversity present in the east remain unclear. Previous work has indicated that the northern episystem is linked to that of island South East Asia and Melanesia, and that BTV present in Indonesia, Papua New Guinea and East Timor, may act as source populations for new serotypes and genotypes of BTV to enter Australia’s north. In this study, the genomes of 49 bluetongue viruses from the eastern episystem and 13 from Indonesia were sequenced and analysed along with 27 previously published genome sequences from the northern Australian episystem. The results of this analysis confirm that the Australian BTV population has its origins in the South East Asian/Melanesian episystem, and that incursions into northern Australia occur with some regularity. In addition, the presence of limited genetic diversity in the eastern episystem relative to that found in the north supports the presence of substantial, but not complete, barriers to gene flow between the northern and eastern Australian episystems. Genetic bottlenecks between each successive episystem are evident, and appear to be responsible for the reduction in BTV genetic diversity observed in the north to south–east direction.

**Electronic supplementary material:**

The online version of this article (10.1186/s13567-017-0488-4) contains supplementary material, which is available to authorized users.

## Introduction

Bluetongue virus (BTV) (genus *Orbivirus*, family *Reoviridae*) is a significant livestock pathogen that is transmitted primarily by biting midges (*Culicoides* spp.) and occurs on all continents except Antarctica [1, 2]. Disease caused by BTV can be severe, mainly affecting sheep, goats and some wild ruminants, whilst cattle act as reservoirs of infection but are usually asymptomatic, with some notable exceptions [1, 3, 4]. However, variations in pathogenicity have been observed amongst both the 27 known and potentially new BTV serotypes, and the strains within these serotypes, with some causing only mild disease and others resulting in severe pathology [3, 5, 6]. The global annual economic cost of BTV infection in livestock has been estimated to be as much as US $3 billion from lost production and disruptions to international trade, making it one of the most economically significant pathogens of livestock [5, 7].

BTV virions are double-shelled particles comprising 7 proteins (VP1–VP7) and a double-stranded (ds) RNA genome comprising 10 segments (Seg-1–Seg-10) [8]. Of these, the highly variable outer capsid protein VP2 (encoded by Seg-2) is of particular importance as it is the primary factor in determining the virus serotype (with potential contributions from VP5/Seg-6 for some serotypes) [9, 10]. Novel BTVs are often the result of genome segment reassortment, which is a regular consequence of mixed BTV infection. This can occur either in the vector or vertebrate host, and within and between serotypes [11–13]. Reassortment provides a mechanism for rapid evolution of the virus and shapes the ecology of bluetongue disease [11–13, 16].

The global distribution of BTV varies with climate, occurring predominantly in tropical and subtropical areas with recent extensions into temperate zones, and is limited primarily by the presence of suitable vectors [1]. Geographical variation in the distribution of BTV serotypes/strains and associated vector species has resulted in several distinct, but partially overlapping, epidemiological systems (episystems) in most regions with significant BTV infection, including Europe, North America and Asia [14, 15]. For most of these episystems, the underlying evolutionary and epidemiological dynamics are poorly understood, hindering efforts to control or eradicate the virus.

In Australia, the distribution of BTV is represented by two episystems: one in the Northern Territory (NT) and adjacent regions of Western Australia (i.e., the northern episystem) and one in the eastern states of Queensland (QLD) and New South Wales (NSW) (i.e., the eastern episystem) [16–18]. The northern episystem may also be connected to a larger and more diverse episystem that encompasses the neighbouring islands of South East Asia and Melanesia (henceforth referred to as the island South East Asian and Melanesian, or SEAM, episystem) [17, 18]. BTV was first detected in Australia in the NT in 1975, and intensive surveillance efforts since have resulted in the identification of 12 serotypes in the northern episystem (BTV-1, 2, 3, 5, 7, 9, 12, 15, 16, 20, 21 and 23), with a subset of these (BTV-1, 2, 15 and 21) also present in the eastern episystem [16, 19, 20]. Despite the presence of some pathogenic strains of BTV in Australia, bluetongue disease has not yet been reported, which may be due to the limited overlap between sheep farming regions and those where BTV is prevalent. The introduction of new BTV serotypes and genotypes into Australia is thought to occur via the northern episystem through the wind-borne dispersal of infected midges from neighbouring islands, ranging from the Indonesian Archipelago east of Lombok, across southern Papua New Guinea, including the island of Timor [21–23]. However, only a small subset of the antigenic diversity entering the NT has filtered into the eastern episystem, despite the lack of obvious biological or ecological barriers to dispersal [16, 23]. Although this suggests that the northern episystem may be the source of BTV genetic diversity in eastern Australia (with only a subset of this diversity establishing in the eastern episystem), the dynamics that govern the interactions between Australian BTV populations are not understood. In this paper, we examine the genetic structure of the northern and eastern Australian BTV episystems in the context of viruses found in nearby Indonesia (representing the SEAM BTV population), and show that while the northern episystem appears tightly linked with that of SEAM, the eastern episystem has remained relatively distinct and stable over time. Notably, we were able to detect only four separate incursions of BTV into the eastern episystem over a 31-year period, although further sampling may reveal additional BTV movement between the northern and eastern episystems.

## Materials and methods

### Genome sequence determination

To begin to understand the links between the northern and eastern Australian BTV episystems, 49 viruses assigned antigenically as BTV-1 (*N* = 12 from QLD, *N* = 20 from NSW), BTV-2 (*N* = 3 from QLD), BTV-15 (*N* = 1 from QLD), and BTV-21 (*N* = 5 from QLD, *N* = 8 from NSW) were selected for full genome sequencing (Additional file 1, Figure 1). Thirteen viruses representing BTV-3, 9, 16, 21 and 23 from Indonesia (sampled between 1988 and 1991) were also sequenced to explore the relationship between the viruses circulating in the SEAM episystem and those that have appeared in Australia.

**Figure 1:**
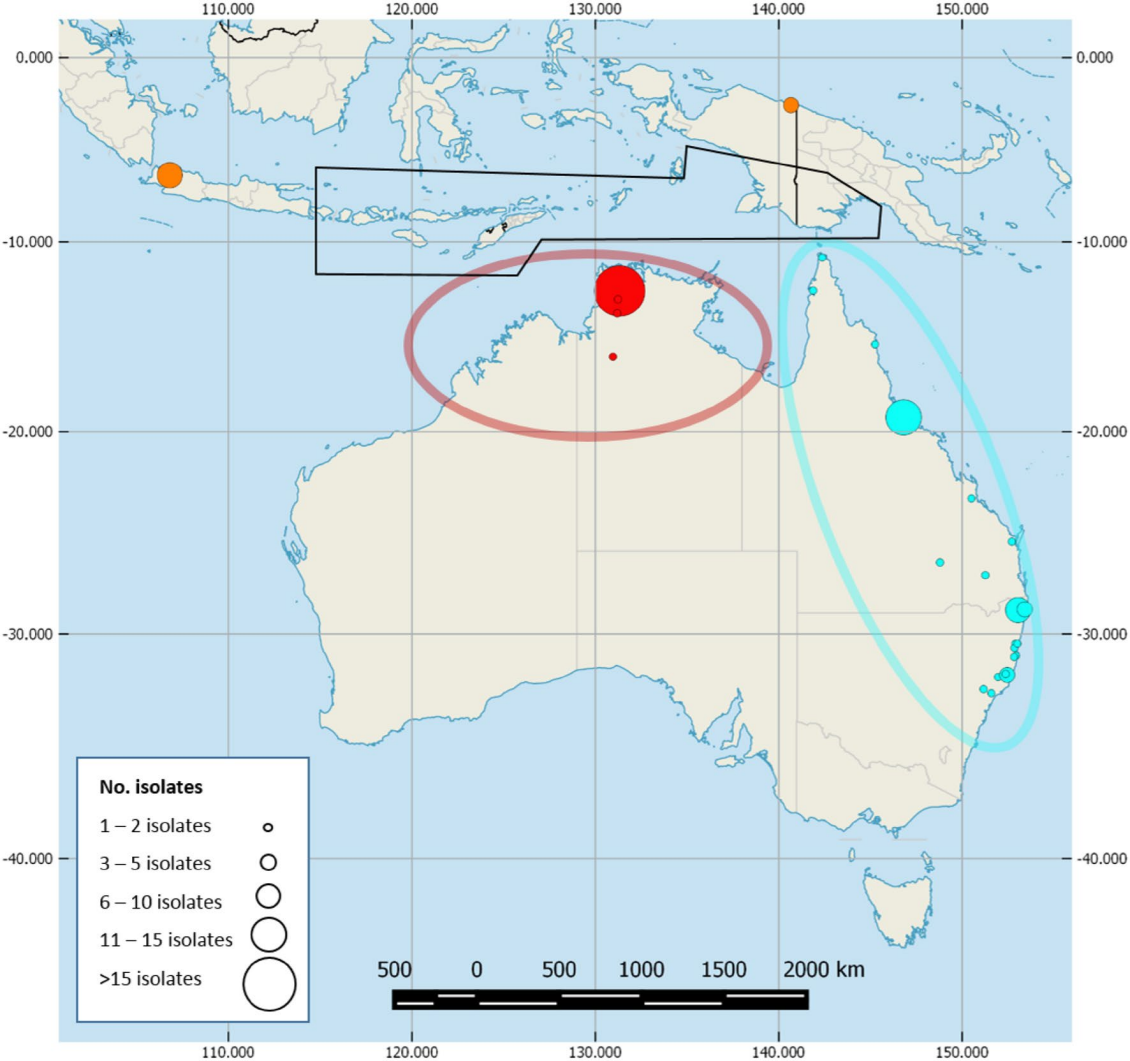
**Sites of collection of the BTV genomes analysed in this study.** The geographical boundaries of the northern (red) and eastern (blue) Australian episystems are shown along with the location of the samples sequenced in this study. Samples from the northern Australian episystem are shown in red, those from the eastern episystem in blue, and those from Indonesia are indicated in orange. The number of isolates is indicated by the size of circle at each location. The epidemiologically predicted island dispersal area for BTV-infected *Culicoides* entering Australia is indicated by a black polygon.

BTVs were cultured from frozen aliquots kept from the original isolations, which were conducted using one or more of: embryonated chicken eggs, baby hamster kidney 21 (BHK-21) cells, and *Aedes albopictus* cells (C6/36). Each virus was cultured for sequencing using a single 75-cm^2^ flask of the BSR clone of BHK-21 cells at 70–80% cell confluence, and Eagle’s basal medium (BME) supplemented with 5% fetal bovine serum at 37 °C. Viral preparations were harvested once cytopathic effects were well progressed. BTV dsRNA was purified and prepared for sequencing using the methods of Boyle et al. [17], followed by DNase digestion using Turbo DNase (Ambion), and cDNA synthesis using Superscript III (Invitrogen), with 50 ng of random hexamers and 0.5 ng of primers specific to the ends of the BTV genome segments (5′ GTTAAAN 3′ and 5′ GTAAGTN 3′) [17]. Following digestion with RNase H (Invitrogen), dsDNA was prepared by treating the cDNA with Klenow Fragment (NEB), purified using the Qiagen PCR purification kit, and prepared for sequencing using the Nextera XT DNA library preparation protocols and adaptors (Illumina), following the manufacturers specifications. Paired-end, 250- or 350-base-read protocols were used for sequencing on an Illumina MiSeq instrument.

BTV genomes were assembled using a combination of de novo assembly and mapping to BTV reference genomes using CLC Genomics Workbench version 6. Assemblies were curated manually and edited using Geneious version 9.1 [24], and consensus sequences were generated for each segment. The consensus sequences were deposited in GenBank (Accession Numbers KY485039-KY485100, MF384442-MF384999).

### Phylogenetic analyses

All available full genome sequences of BTV were downloaded from GenBank and combined with those generated in this study. Genomes for which country or year of sampling could not be obtained were removed from the dataset, resulting in 272 full genome sequences from 39 countries, covering the years 1969–2013. The northern Australian episystem was represented by 27 genomes from 10 serotypes (*N* = 18 BTV-1, *N* = 1 of each of BTV-2, 3, 7, 9, 15, 16, 20, 21, 23) collected between 1977 and 2010 [16, 17]. For each segment, sequences were trimmed to include only the complete coding region and aligned using CLUSTALW [25] with manual refinement. Each alignment was screened for recombination using the SBP and GARD tools available in the HyPhy package with the best-fit nucleotide substitution model determined using the model selection procedures also available within HyPhy [26]. No significant evidence was found for recombination.

Maximum likelihood phylogenetic trees were estimated in PhyML version 3.0 [27], employing the best-fit nucleotide substitution model for each segment, determined as above, and subtree pruning and regrafting branch-swapping. The phylogenetic robustness of each node was determined using 1000 bootstrap replicates. Cluster Picker version 1.2 [28] was used to define monophyletic clades with bootstrap support values of 0.85 and a genetic distance threshold for clusters of 5%, as in previous work [12]. Clusters were used to assign each virus to a genotype at each segment.

## Results

### Genetic diversity of BTV in Indonesia and Australia’s Northern Territory

**Figure 2:**
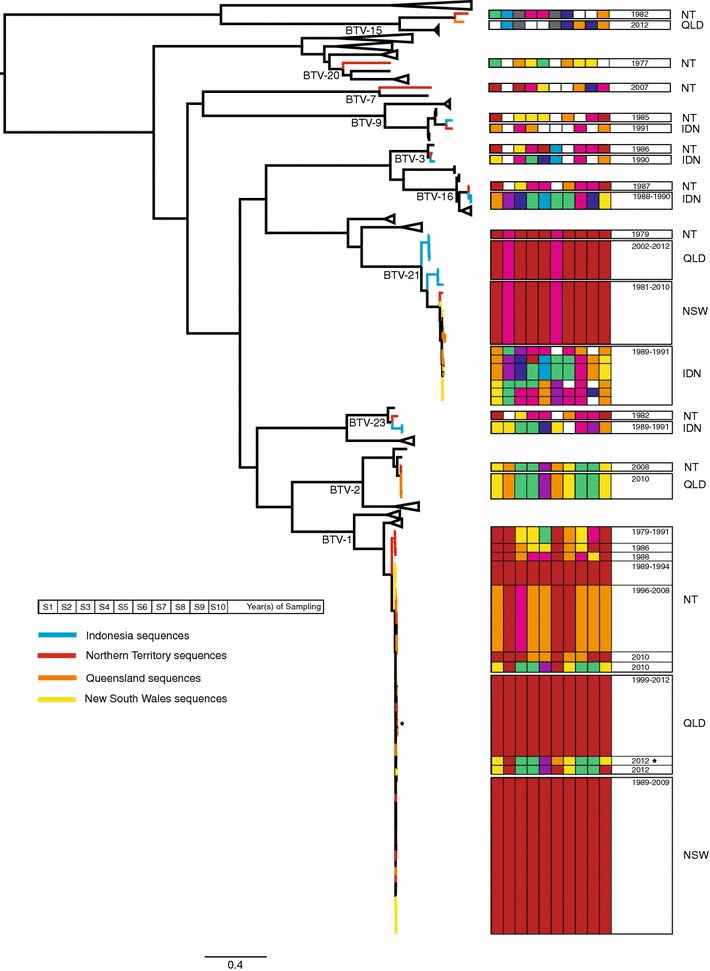
**Phylogeny of BTV Seg-2 and associated genome segment constellations.** Maximum likelihood tree of 272 viruses from 30 countries, with clades not containing viruses from Indonesia or Australia collapsed for visual clarity. Viruses were assigned to clades for each genome segment using Cluster Picker with bootstrap support values > 0.85 and a genetic distance threshold of 5% [25]. Sequences from the same segment that were assigned to the same cluster are indicated by shared colours in the displayed genome segment constellations. The size of each genome constellation is proportional to the number of isolates with that genome. The position of BTV-1 isolate: 2012-26 is indicated by a star.

The full genomes of 13 viruses assigned to BTV-3, 9, 16, 21 and 23 from Indonesia were sequenced in this study, revealing a surprising amount of genetic diversity for most genome segments (Additional files 1, 2, Figure 2). All seven of the BTV-21 viruses sequenced here were collected between 1989–1991, yet between two and five distinct genotypes were present at each segment within the serotype, suggesting that a diverse group of BTVs may circulate in Indonesia and the islands of SEAM at any one time (Figure 2). Furthermore, whilst the Indonesian viruses clustered within larger clades that often included viruses from across Asia, they were more closely related to viruses from Australia than they were to those circulating in China, India or Taiwan, irrespective of the segment analysed (Additional file 2). As has been observed in other regions, reassortment has played an important role in shaping the genomic diversity present in Indonesia [11, 12, 17]. Our phylogenetic analysis reveals multiple instances where viruses assigned to different serotypes share nearly identical genome segments (Figure 2).

**Figure 3:**
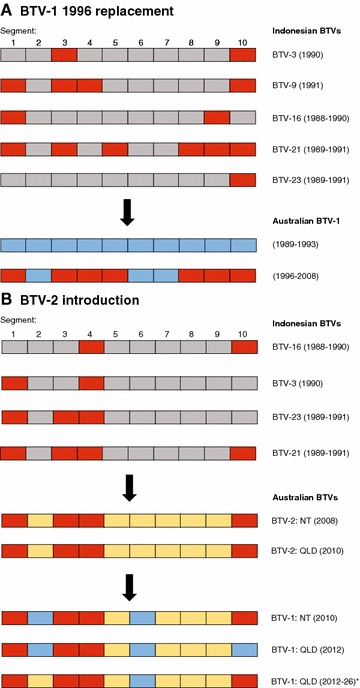
**Genome segment sharing between Indonesian and northern Australian BTV isolates.** Year(s) of circulation are shown in parentheses and presumed reassortment events are indicated with an arrow. **A** The replacement of the existing Australian BTV-1 lineage from 1989 to 1993 (blue blocks) with segments circulating in concurrent Indonesian viruses (red blocks) is shown. **B** The introduction of BTV-2 into Australia and subsequent reassortment with BTV-1 viruses is shown. BTV-2 segments also circulating in concurrent Indonesian BTVs are indicated in red, segments unrelated to others in our dataset are shown in yellow, and pre-existing Australian BTV-1 segments are indicated in blue.

Although spatial modelling of aerial displacement of *Culicoides* midges suggests that Indonesia, East Timor or New Guinea may be a significant source population of Australian BTV [21], support for this hypothesis has been limited by the absence of complete genome sequences from viruses in this region [18]. Despite sequencing only 13 Indonesian virus genomes in this study, it is clear that the Indonesian/SEAM and northern Australian episystems are tightly linked (Figure 2). The phylogenetic and temporal relationships between the NT and Indonesian viruses reveal that each new incursion of BTV into the northern Australian episystem introduced novel genome segments that were previously or concurrently a part of the diversity circulating in Indonesia. For example, it has been noted that BTV-1 evolution in the NT underwent a period of relative stasis from 1989 to 1994, with a “genomic backbone” (i.e., the non-serotype determining segments: 1, 3, 4, 5, 7, 8, 9, and 10) shared between the dominant BTV-1 genotype and the BTV-21 viruses that were also circulating in the north (Figure 2) [16]. Beginning in 1996, a reassortment event led to the complete replacement of this 1989-era BTV-1 genotype with a virus containing seven novel genome segments that had not been detected previously in Australia. The results of our analysis clearly show that each of these segments is closely related to those found in Indonesian viruses during this period (1988–1991): Seg-1 was detected in Indonesian BTV-9, 16 and 21; Seg-3 in BTV-3, 9 and 21; Seg-4 in BTV-9; Seg-5 in BTV-21; Seg-8 in BTV-21; Seg-9 in BTV-16 and 21; and Seg-10 in BTV-3, 9, 21 and 23 (Figure 3A). Similarly, the constellation of genome segments that was first detected in Australia with the arrival of BTV-2 in 2008 and quickly reassorted with Seg-2 and 6 of BTV-1 [16] clearly shows relationships with viruses circulating earlier in Indonesia (1988–1989): Seg-1 was detected in Indonesian BTV-3, 21, and 23; Seg-3 in BTV-21 and 23; Seg-4 in BTV-3, 16, 21 and 23; and Seg-10 in BTV-16 and 21 (Figures 2 and 3B). Although no Indonesian BTVs isolated after 1991 were available for sequencing, the timing of the incursion of BTV-2 into Australia suggests that many of these segments must have still been circulating in the SEAM episystem in 2008.

### The eastern Australian episystem

To characterize the genetic diversity within the eastern Australian episystem, the full genomes of viruses assigned antigenically to BTV-1 (*N* = 20 from NSW, *N* = 12 from QLD), BTV-2 (*N* = 3 from QLD), BTV-15 (*N* = 1 from QLD), and BTV-21 (*N* = 8 from NSW, *N* = 5 from QLD) were sequenced and analysed along with a previously published BTV-2 genome from QLD (Additional files 1 and 2). In contrast to the high levels of genetic diversity present in the northern Australian and SEAM episystems, the eastern Australian episystem was characterised by extreme genetic homogeneity that has persisted both through time and across the entire eastern range of BTV (Figure 2). All BTV-1 and BTV-21 viruses sampled during the 1981–2012 period carried the same genomic backbone that was present in BTV-21 in the NT from at least 1979, and in BTV-1 in the NT from 1989 (Figure 2). It is striking that despite the continued introduction and reassortment of new genome segments into the northern episystem, none of this diversity was detected in viruses from QLD or NSW until the introduction of BTV-2, when a significant shift in the circulating diversity was observed across eastern Australia.

BTV-2 was detected for the first time in the NT in 2008, and 2 years later was isolated in sentinel cattle in QLD [29]. At this time (2010), the BTV-2 genomic backbone (including Seg-6) appeared in BTV-1 isolates in the NT, and by 2012 this constellation of segments had also appeared in BTV-1 isolates from QLD, where it co-circulated with the dominant BTV-1 genotype (Figure 3B). Subsequent inter-serotype reassortment within the eastern Australian episystem also occurred, as we identified a virus isolate from QLD that had been serologically typed as BTV-15, but clustered well within a group of BTV-1 sequences from QLD in the Seg-2 phylogeny (hereafter referred to as BTV-1 isolate: 2012-26) (Figure 2). However, based on the phylogenetic position of isolate 2012-26 in the trees constructed from the remaining segments, it’s clear that this virus has the BTV-2 genomic backbone (Figure 3B). The reassortment event that led to this virus carrying Seg-2 from the QLD BTV-1 population and a BTV-2 genomic backbone may have complicated serotype determination using traditional serological methods, leading to the mistyping of this virus as BTV-15. Alternatively, the original sample from which both the serotype and genome sequence were determined may have contained multiple co-infecting viruses belonging to BTV-15 and the BTV-1/BVT-2 reassortant. Additional work is underway to clarify the antigenic classification of this isolate and its relationship to the sequence reported here. In contrast to the recent shifts that have occurred in the evolution of BTV-1 in the eastern Australian episystem, the genomic backbone found in viruses assigned as BTV-21 in QLD and NSW remained unchanged across the entire sampled period (1981–2012) (Figure 2).

## Discussion

This study provides the first detailed assessment of the genetic diversity in, and relationships between, the northern and eastern Australian BTV episystems in the context of viruses from the Indonesian Archipelago, a close neighbour to Australia’s north. Although the northern and eastern Australian episystems are clearly linked to each other, as well as to the larger SEAM episystem (represented here only by Indonesia), they also appear to be operating under distinct evolutionary and ecological constraints that warrant further investigation.

### Indonesia and the northern Australian episystem

The results of this study support previous suggestions that BTV periodically enters Australia via the NT or WA from one or more regions off the northern coast, including those belonging to Indonesia, Papua New Guinea and East Timor [21, 22]. Indeed, the genetic diversity present in the NT BTV population at any one time appears to be a subset of the diversity present in the concurrent or historical Indonesian population. This suggests that the relationship between the Australian BTV population and that of neighbouring landmasses may be best characterised by a source-sink epidemiological model, where instances of viral movement occur primarily (but not exclusively) in the north–south direction [21, 30–32]. *Culicoides* populations in Australia appear to be derived from those found across the islands of South East Asia, and evidence suggests that multiple introductions are likely to have occurred [21, 31]. It is therefore possible for BTV to have entered Australia, not only from the locations studied here, but from across the near islands of South East Asia and Melanesia, as far east as the Indonesian island of Lombok to the Solomon Islands (Figure 1) [21, 31]. Although the results of our phylogenetic analysis generally support this region as the primary source of Australian BTV, the only viruses available for sequencing from Indonesia, East Timor, Papua New Guinea or the Solomon Islands were collected from West Java and northern Papua (previously West Papua). Although these regions are outside the previously modelled wind dispersal area, they do bracket the predicted dispersal zone (Figure 1), and evidence of BTV infection has been detected throughout this region [32–34]. In addition, sequences of multiple segments from Papuan BTV-16, BTV-21 and BTV-23 isolates were closely related to those found in West Java, suggesting that this entire region may have a contiguous BTV population. However, without additional sampling of BTV across all neighbouring landmasses to Australia’s north, it will be impossible to determine if the genetic diversity we observed in our Indonesian isolates is representative of that circulating in the region as a whole (i.e., if this entire region supports a single, mixed virus population), or if there are additional epidemiological dynamics that further constrain virus movement across this region and into Australia. With additional and thorough sampling across all likely source locations, it may be possible to further clarify the origin(s) of BTV in northern Australia.

Active BTV surveillance programs have been in place throughout Australia since 1979, and involve frequent serological testing using an extensive network of sentinel cattle in combination with surveillance for the presence of exotic *Culicoides* species. Studies have shown that this surveillance program may sometimes be effective enough to detect incursions of novel BTV serotypes within a few weeks of arrival [22, 35]. Twelve serotypes have been detected in the NT since 1975, indicating that BTV has successfully entered Australia at least 12 times; however, several serotypes (e.g., BTV-3, BTV-16) have been isolated only sporadically since first detection, with long periods between isolations [16]. This may suggest that local extinction and reintroduction of some serotypes in the north has occurred multiple times, or that these serotypes have persisted at too low a prevalence to be consistently detected in livestock only to re-emerge years later, perhaps when optimal conditions for transmission are present. With the data currently available, it is not possible to discriminate between these two hypotheses, nor is it possible to determine how frequently commonly detected serotypes like BTV-1 or BTV-20 (comprising 55% of all BTV isolations in the NT) re-entered Australia’s north [16]. This is due in part to the limited genetic diversity seen in each individual segment of the sequenced Australian viruses (i.e., there are fewer genotypes than serotypes at each segment). Nevertheless, the substantial contribution of genome segment reassortment cannot be discounted, as this process constantly generates new combinations of existing segments, rapidly obscuring the evolutionary history of each serotype (Figure 3, Additional file 2) [12, 13, 16, 17]. However, because only a very small fraction of BTV genomes have been sequenced from the region (and these are primarily representatives of BTV-1 and BTV-21), we are likely to be underestimating the genetic diversity circulating in both SEAM and Australia, thereby underestimating the number of BTV incursions and the frequency of reassortment in the Australian episystems.

### Stability of the eastern Australian episystem

The distribution and dynamics of BTV in Australia are shaped by several factors that have resulted in the establishment of distinct, yet interconnected episystems in the north and east. The spatiotemporal distribution of suitable vectors is perhaps the most significant of these, and is in turn influenced by temperature, humidity, wind patterns, and the availability of suitable breeding habitat [36, 37]. Vector distribution is well known to be a key factor in determining the spatial distribution of arboviruses globally, and has been implicated in the spatiotemporal patterns of key arboviruses of livestock in Australia, including BTV, bovine ephemeral fever virus (family *Rhabdoviridae*) and Akabane virus (family *Bunyaviridae*) [37, 38]. In Australia, both Akabane virus and BTV are transmitted primarily by biting midges and show highly similar geographic distributions, whereas bovine ephemeral fever virus is primarily vectored by mosquitoes and is more broadly distributed throughout the country in a single episystem [39, 40]. *C. brevitarsis* is considered to be the most significant vector of BTV in Australia and the geographic distribution of BTV closely mirrors that of this midge species, with both vector and virus largely absent from the sheep-rearing regions of southern Australia [23, 30, 41]. The distribution of *C. brevitarsis* across QLD and NSW fluctuates throughout the year with variations in wind and temperature acting to influence both the introduction of midges from northern Australia in the warmer months, and their local extinction during the winter months [42]. Annual incursions of *C. brevitarsis* from the north may therefore provide a conduit that could facilitate the regular introduction of new BTV serotypes and genotypes into the eastern episystem. The links between these midge populations is supported by the lack of genetic differentiation observed between the predominant *C. brevitarsis* populations in the northern and eastern episystems, although recent work has suggested that multiple incursions of *C. brevitarsis* into the east may have also occurred from outside Australia [23, 31, 43]. However, the results of our analysis do not appear to suggest that the movement of BTV from the north to the east occurs with high frequency. Rather, the eastern Australian episystem is characterized by extreme genetic homogeneity that has persisted both through time and across the entire eastern range of BTV, and contrasts with the high levels of genomic diversity observed in the northern episystem. This strongly suggests that only a relatively small number of viruses have successfully invaded eastern Australia since 1979, and these have persisted in an unusually stable episystem ever since. Although the arrival of BTV-2 in the eastern episystem appears to have disrupted this stability to some extent, the factors that facilitated this incursion are unknown, but may be related to favourable climatic conditions (and hence midge distributions) at the time of arrival.

### Bluetongue virus episystems in Australia and Indonesia

Our genetic analyses of the Australian and Indonesian BTV viruses available to date indicate that all sampled BTVs in Australia have their origins in one or more of the island landmasses to the north of the country. Genetic bottlenecks resulting from founder events have limited the genetic diversity present in the NT relative to Indonesia, and similarly reduced further the diversity in the eastern episystem relative to the northern. Successful incursions of BTV into Australia require multiple steps, each of which is governed by several factors that appear to combine to reduce the total number of introductions into the NT. These include the long-distance dispersal of infected midges, the location of and transmission to a susceptible ruminant, and then ongoing transmission to further susceptible hosts via one or more competent vector species in Australia. The forces that act to further restrict viral movement from the northern to eastern episystems are less clear, but may include a combination of environmental, geographic, ecological and climactic factors. However, the recent incursion and reassortment of BTV-2 into the eastern episystem reveals that links between the northern and eastern episystems persist, and that whatever the barriers to regular mixing are, they are also permeable. Critically, more extensive sampling and sequencing of BTVs in Australia and South East Asia will be required before a more comprehensive understanding of the relationships among the South East Asian and Australian episystems can emerge.

## Additional files

**Additional file 1.** **Complete list of Genbank accession numbers for all previously-published sequences of Australian BTVs analysed in this study.**

**Additional file 2.** **Maximum likelihood phylogenetic trees for genome segments 1–10.**
