# Supplementary material for: Genomic analysis of bluetongue virus episystems in Australia and Indonesia
Source: Vet Res. 2017 Nov 23;48:82. doi: 10.1186/s13567-017-0488-4 (PMC5701493; doi:10.1186/s13567-017-0488-4)
Supplement: Supplementary file 1 — Additional file 1. Complete list of Genbank accession numbers for all previously-published sequences of Australian BTVs analysed in this study. [file 13567_2017_488_MOESM1_ESM.pdf]

Complete list of Genbank accession numbers for all previously-published sequences of Australian BTVs analysed in this study.

| Serotype | Accession | Isolate | Country | State | Location | Species | Year | Ref |
|---|---|---|---|---|---|---|---|---|
| BTV-1 | JN881985-<br>JN881994 | CSIRO156 | AUS | NT | Beatrice Hill | Cattle | 1979 | 14 |
| BTV-1 | KM099506<br>KM099523<br>KM099540<br>KM099557<br>KM099574<br>KM099591<br>KM099608<br>KM099625<br>KM099624<br>KM099659 | DPP0065 | AUS | NT | Beatrice Hill | Cattle | 1981 | 13 |
| BTV-1 | KM099507<br>KM099524<br>KM099541<br>KM099558<br>KM099575<br>KM099592<br>KM099609<br>KM099626<br>KM099643<br>KM099660 | DPP1000 | AUS | NT | Beatrice Hill | Cattle | 1986 | 13 |
| BTV-1 | KM099508<br>KM099525<br>KM099542<br>KM099559<br>KM099576<br>KM099593<br>KM099610<br>KM099627<br>KM099644<br>KM099661 | DPP1315 | AUS | NT | Beatrice Hill | Cattle | 1988 | 13 |
| BTV-1 | KM099509<br>KM099526<br>KM099543<br>KM099560<br>KM099577<br>KM099594<br>KM099611<br>KM099628<br>KM099645<br>KM099662 | DPP1523 | AUS | NT | Beatrice Hill | Cattle | 1989 | 13 |
| BTV-1 | KM099510<br>KM099527<br>KM099544<br>KM099561<br>KM099578<br>KM099595<br>KM099612<br>KM099629<br>KM099646<br>KM099663 | DPP2559 | AUS | NT | Beatrice Hill | Cattle | 1993 | 13 |
| BTV-1 | KM099511<br>KM099528<br>KM099545<br>KM099562<br>KM099579<br>KM099596 | DPP3072 | AUS | NT | Beatrice Hill | Cattle | 1994 | 13 |

| | | | | | | | | |
|---|---|---|---|---|---|---|---|---|
| | KM099613<br>KM099630<br>KM099647<br>KM099664 | | | | | | | |
| BTV-1 | KM099512<br>KM099529<br>KM099546<br>KM099563<br>KM099580<br>KM099597<br>KM099614<br>KM099631<br>KM099648<br>KM099665 | DPP4032 | AUS | NT | Beatrice Hill | Cattle | 1996 | 13 |
| BTV-1 | KM099513<br>KM099530<br>KM099547<br>KM099564<br>KM099581<br>KM099598<br>KM099615<br>KM099632<br>KM099649<br>KM099666 | DPP4100 | AUS | NT | Beatrice Hill | Cattle | 1997 | 13 |
| BTV-1 | KM099514<br>KM099531<br>KM099548<br>KM099565<br>KM099582<br>KM099599<br>KM099616<br>KM099633<br>KM099650<br>KM099667 | DPP4588 | AUS | NT | Beatrice Hill | Cattle | 1999 | 13 |
| BTV-1 | KM099515<br>KM099532<br>KM099549<br>KM099566<br>KM099583<br>KM099600<br>KM099617<br>KM099634<br>KM099651<br>KM099668 | DPP4690 | AUS | NT | Beatrice Hill | Cattle | 1999 | 13 |
| BTV-1 | KM099516<br>KM099533<br>KM099550<br>KM099567<br>KM099584<br>KM099601<br>KM099618<br>KM099635<br>KM099652<br>KM099669 | DPP5775 | AUS | NT | Beatrice Hill | Cattle | 2002 | 13 |
| BTV-1 | KM099517<br>KM099534<br>KM099551<br>KM099568<br>KM099585<br>KM099602<br>KM099619<br>KM099636<br>KM099653<br>KM099670 | DPP5844 | AUS | NT | Beatrice Hill | Cattle | 2002 | 13 |

| | | | | | | | | |
|---|---|---|---|---|---|---|---|---|
| BTV-1 | KM099518<br>KM099535<br>KM099552<br>KM099569<br>KM099586<br>KM099603<br>KM099620<br>KM099637<br>KM099654<br>KM099671 | DPP6112 | AUS | NT | Beatrice Hill | Cattle | 2004 | 13 |
| BTV-1 | KM099519<br>KM099563<br>KM099553<br>KM099570<br>KM099587<br>KM099604<br>KM099621<br>KM099638<br>KM099655<br>KM099672 | DPP6504 | AUS | NT | Beatrice Hill | Cattle | 2005 | 13 |
| BTV-1 | KM099520<br>KM099537<br>KM099554<br>KM099571<br>KM099588<br>KM099605<br>KM099622<br>KM099639<br>KM099656<br>KM099673 | DPP7137 | AUS | NT | Beatrice Hill | Cattle | 2008 | 13 |
| BTV-1 | KM099521<br>KM099538<br>KM099555<br>KM099573<br>KM099589<br>KM099606<br>KM099623<br>KM099640<br>KM099657<br>KM099674 | DPP8086 | AUS | NT | Beatrice Hill | Cattle | 2010 | 13 |
| BTV-1 | KM099522<br>KM099539<br>KM099556<br>KM099573<br>KM099590<br>KM099607<br>KM099624<br>KM099641<br>KM099658<br>KM099675 | DPP8304 | AUS | NT | Beatrice Hill | Cattle | 2010 | 13 |
| BTV-2 | JQ086241-<br>JQ086250 | V7291 | AUS | NT | Douglas Daly | Cattle | 2008 | 14 |
| BTV-2 | JQ240321-<br>JQ240330 | Cooktown | AUS | QLD | Cooktown | Cattle | 2010 | 14 |
| BTV-3 | JQ086281-<br>JQ086290 | DPP973 | AUS | NT | Beatrice Hill | Cattle | 1986 | 14 |
| BTV-7 | JQ086291-<br>JQ086300 | DPP6963 | AUS | NT | Beatrice Hill | Cattle | 2007 | 14 |
| BTV-9 | JQ086301 | DP0837 | AUS | NT | Beatrice Hill | Cattle | 1985 | 14 |
| BTV-15 | JQ086221-<br>JQ086230 | DPP192 | AUS | NT | Beatrice Hill | Cattle | 1982 | 14 |
| BTV-16 | JQ086231-<br>JQ086240 | DPP965 | AUS | NT | Beatrice Hill | Cattle | 1987 | 14 |

| | | | | | | | | |
|---|---|---|---|---|---|---|---|---|
| BTV-20 | JQ086251-JQ086260 | CSIRO19 | AUS | NT | Beatrice Hill | Culicoides spp. | 1977 | 14 |
| BTV-21 | JQ086261-JQ086270 | DPP86 | AUS | NT | Victoria River Research Station | Cattle | 1979 | 14 |
| BTV-23 | JQ086271-JQ086280 | DPP90 | AUS | NT | Tortilla Flats Research Farm | Cattle | 1982 | 14 |
