# Supplementary figures and images for: Genomic analysis of bluetongue virus episystems in Australia and Indonesia
Source: Vet Res. 2017 Nov 23;48:82. doi: 10.1186/s13567-017-0488-4 (PMC5701493; doi:10.1186/s13567-017-0488-4)

Segment 1

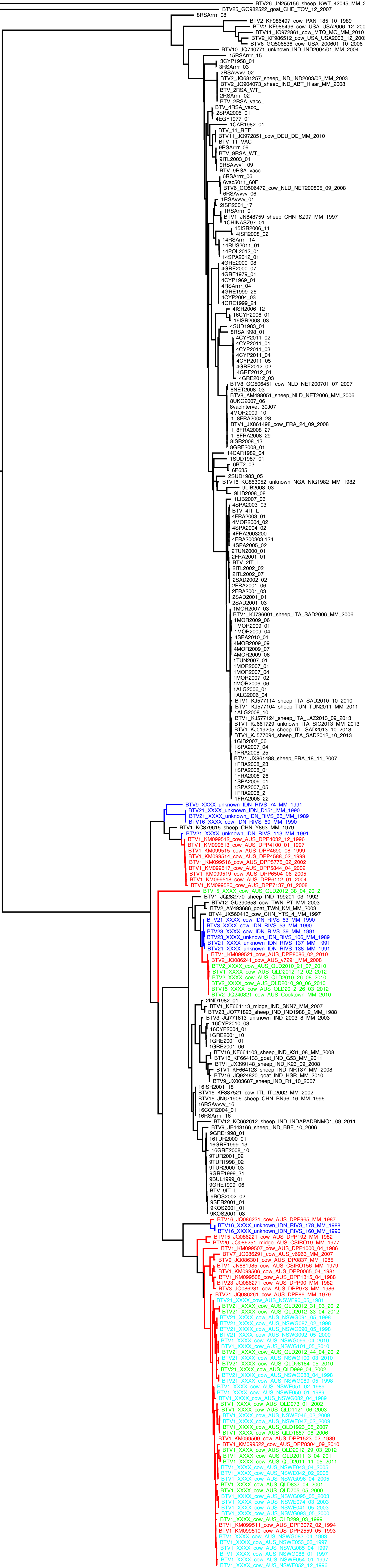

Segment 2

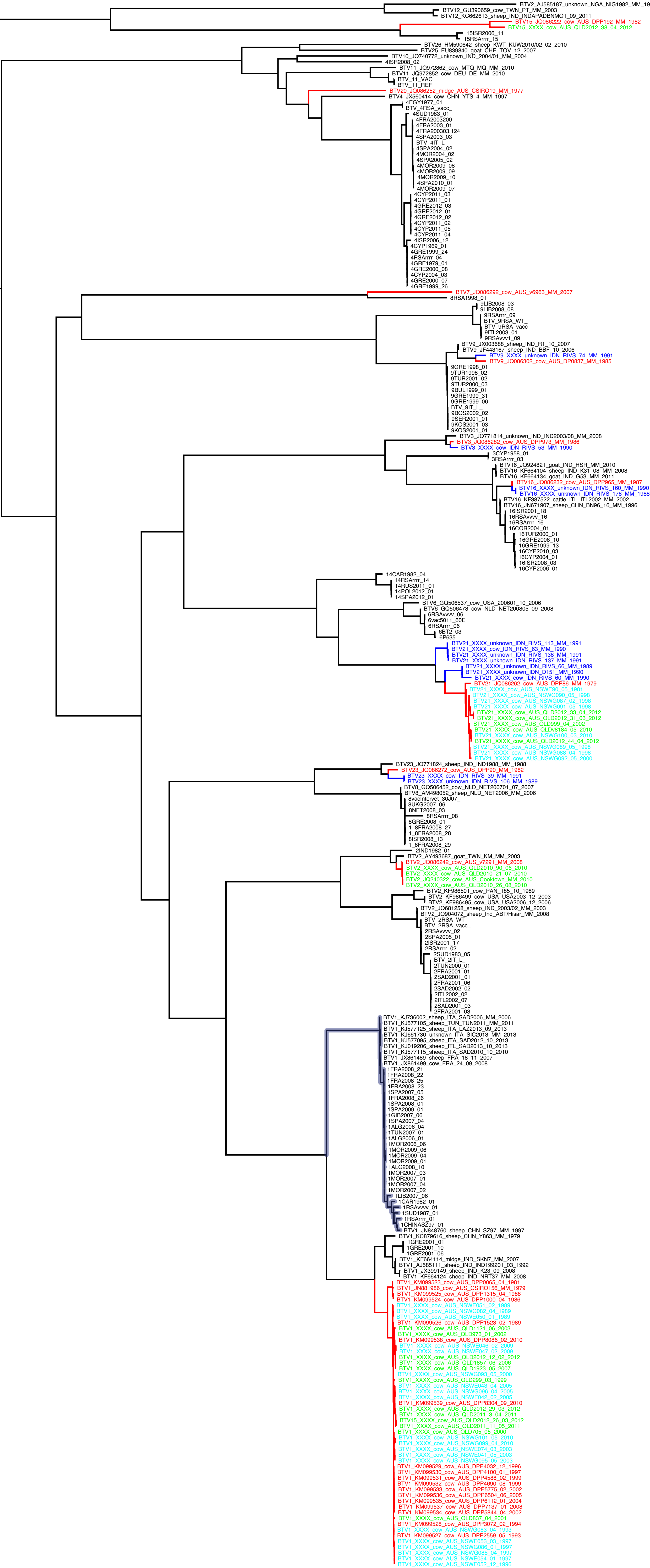

Segment 3

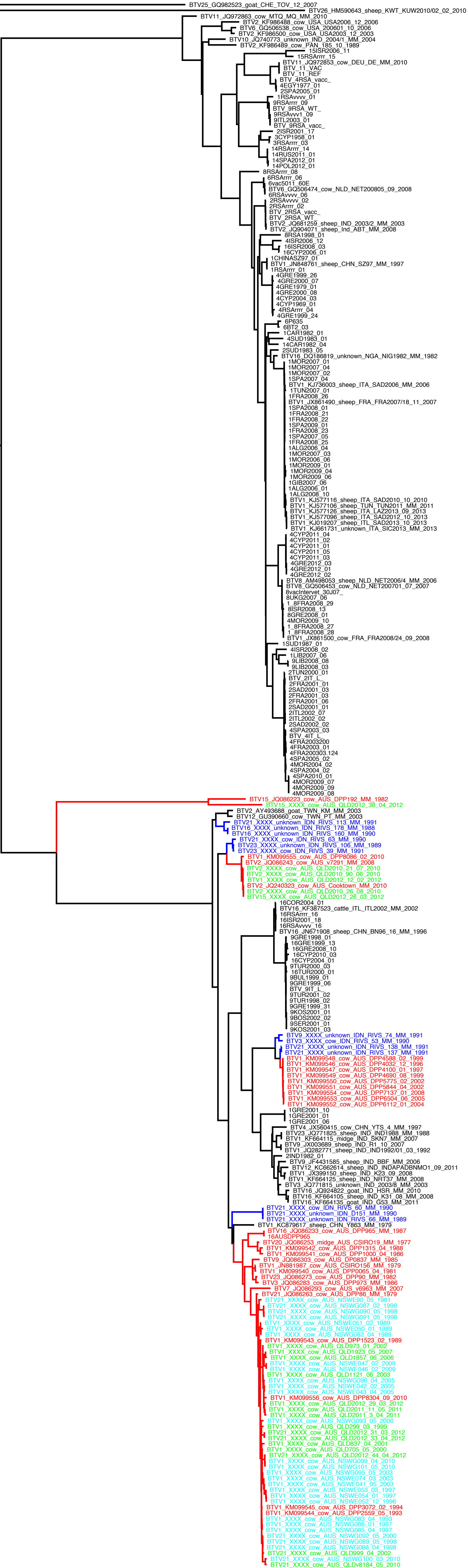

Segment 4

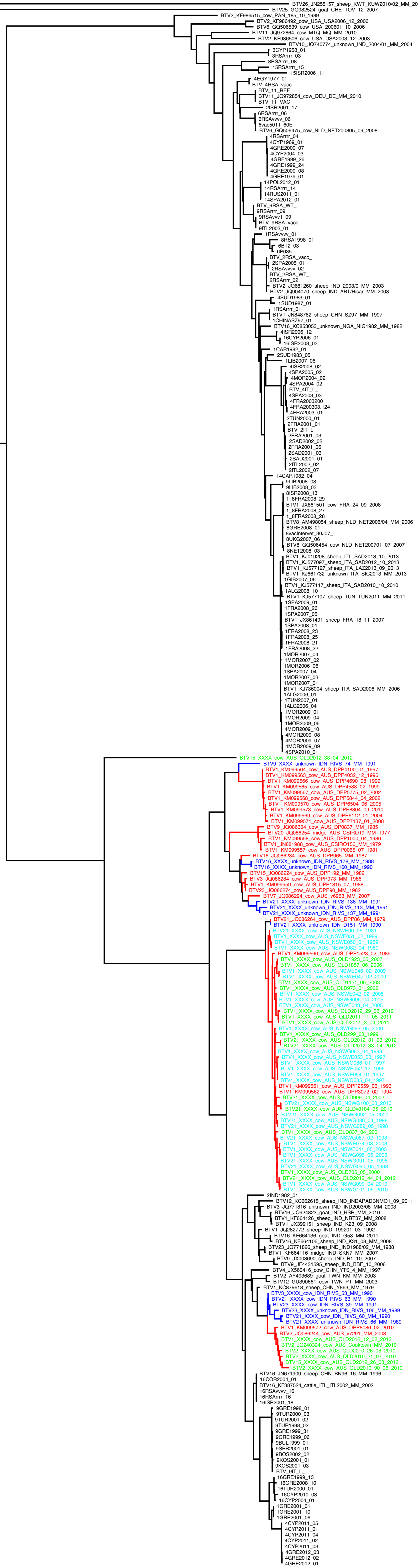

Segment 5

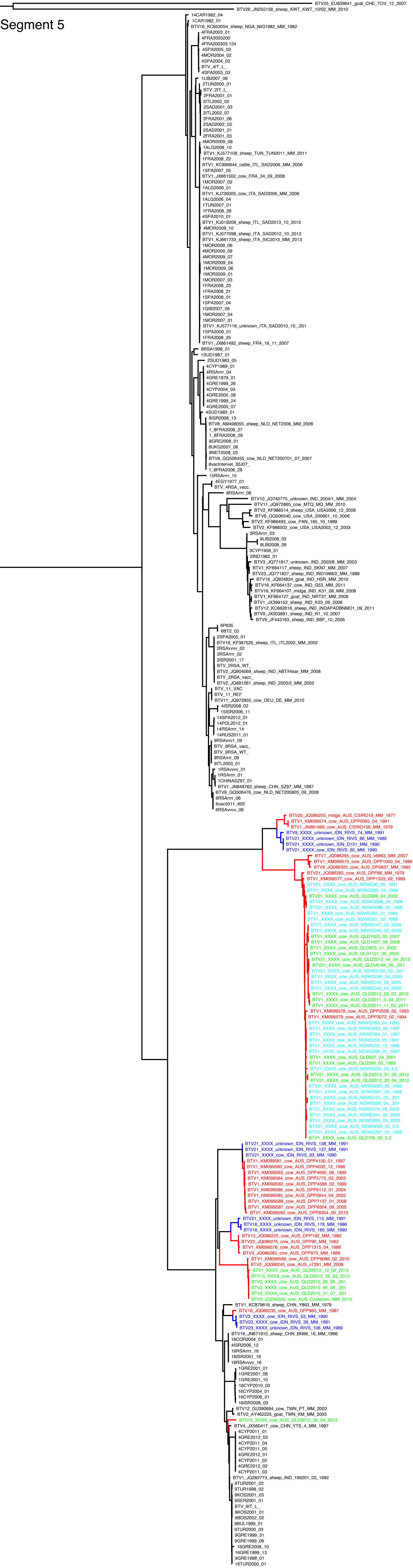

Segment 6

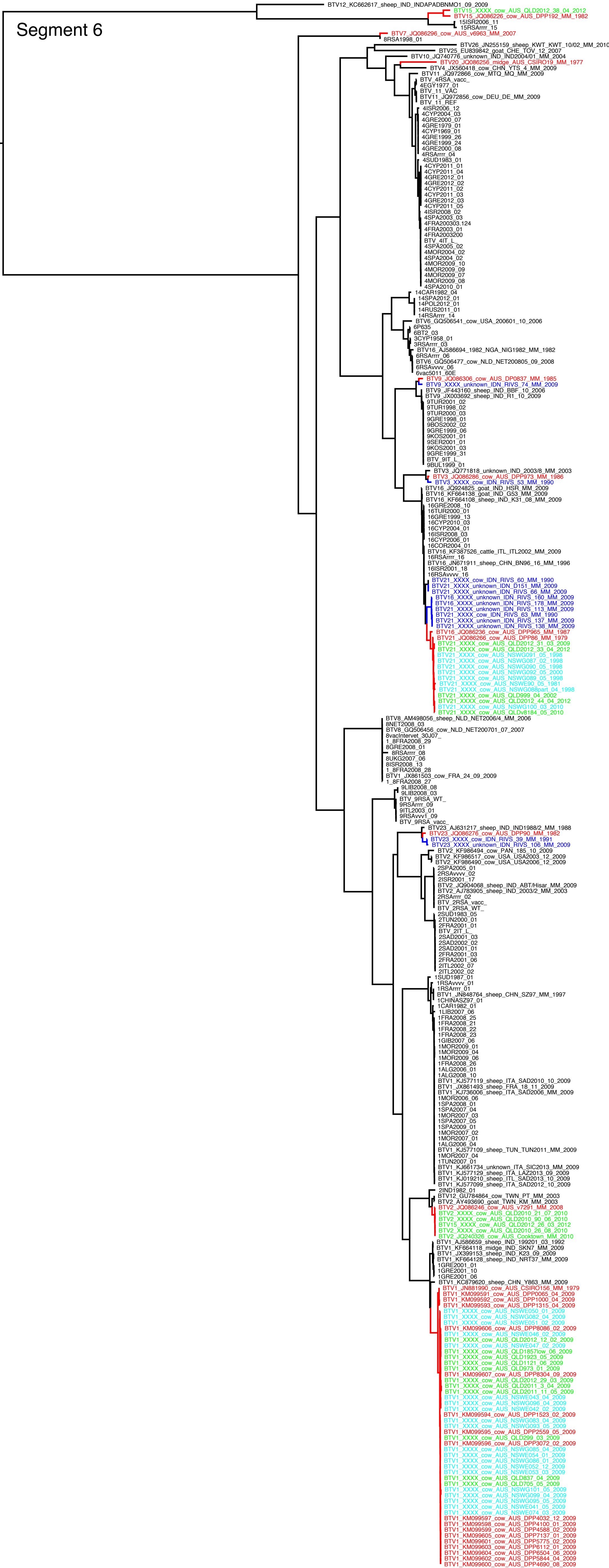

## Segment 7

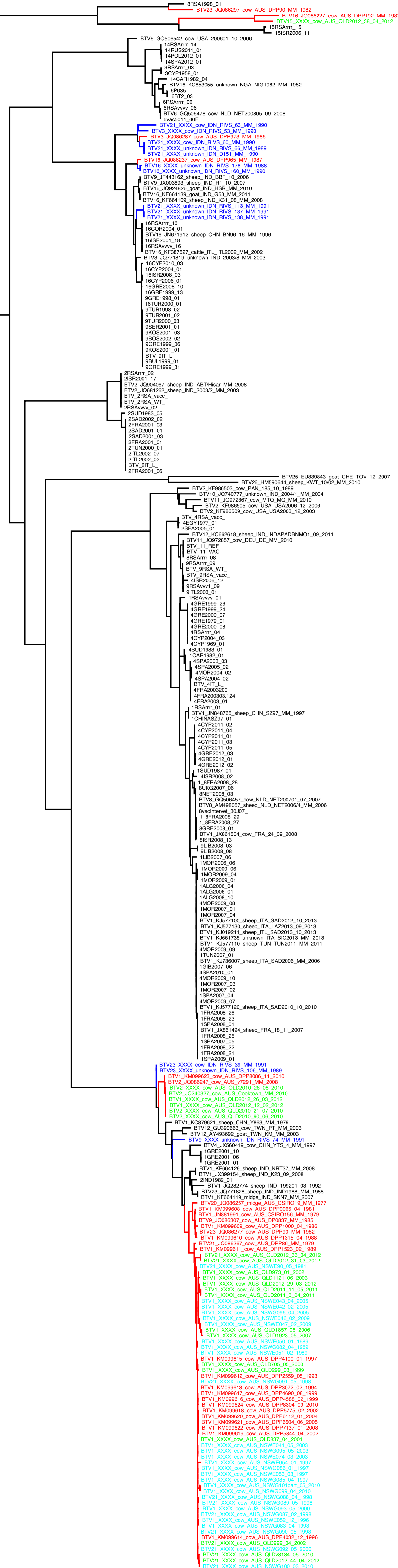

## Segment 8

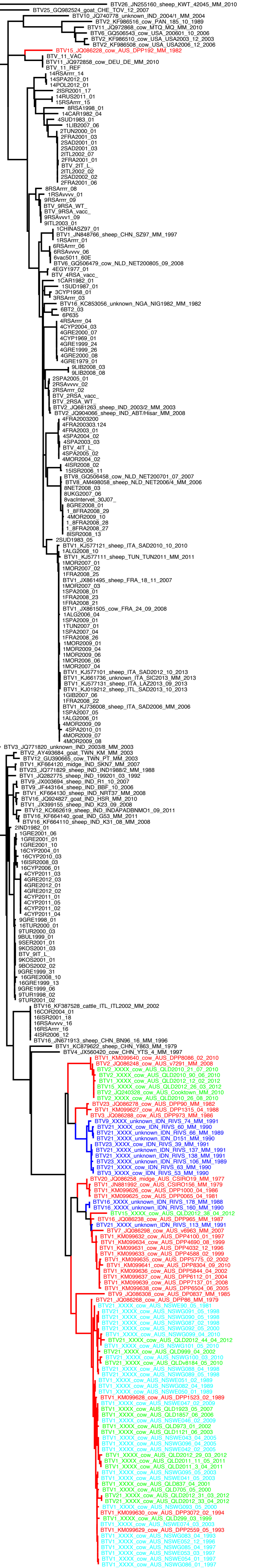



Segment 10

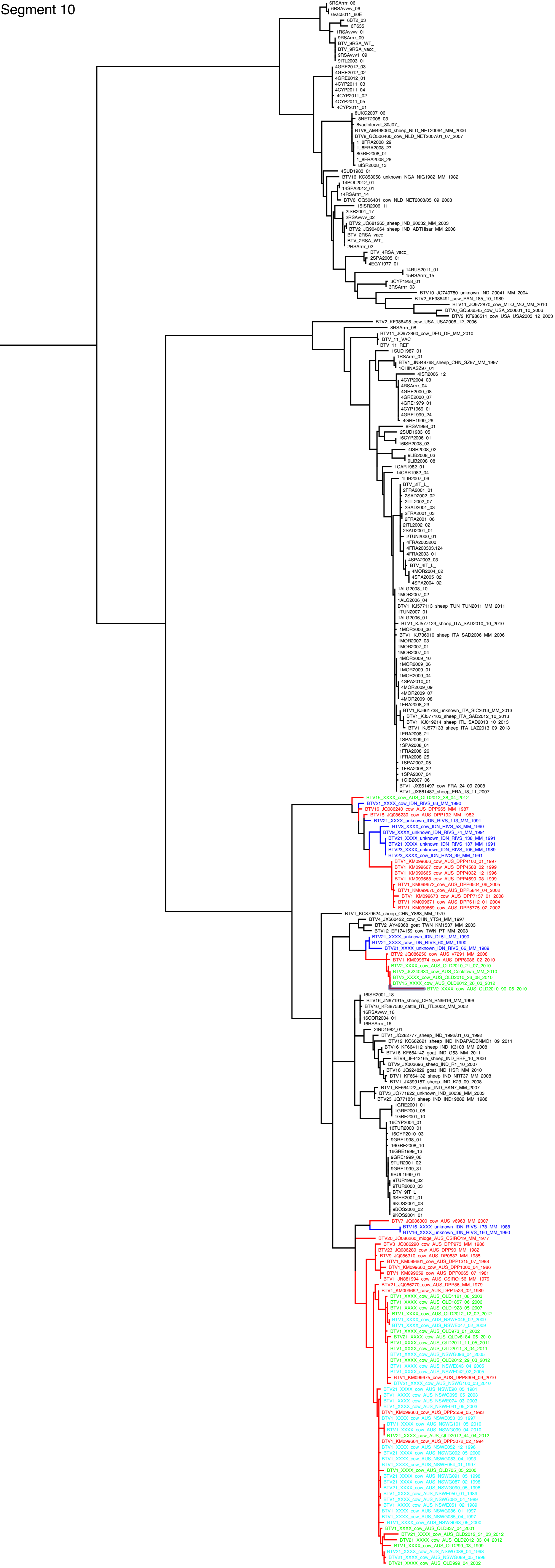

Supplement: Supplementary file 2 — Additional file 2. Maximum likelihood phylogenetic trees for genome segments 1–10. [file 13567_2017_488_MOESM2_ESM.pdf]
